# Potential Soil Contamination in Areas Where Ferronickel Slag Is Used for Reclamation Work

**DOI:** 10.3390/ma7107157

**Published:** 2014-10-23

**Authors:** Seong Seung Kang, Kyungho Park, Daehyeon Kim

**Affiliations:** Department of Civil Engineering, Chosun University, Gwangju 501-759, Korea; E-Mails: kangss@chosun.ac.kr (S.S.K.); munhakng@gmail.com (K.P.)

**Keywords:** FNS (ferronickel slag), engineering properties, soil contamination analysis, natural aggregate

## Abstract

This study aims to analyze contamination with the use of soil reclaimed with ferronickel slag (FNS). In order to investigate any contamination due to FNS disposal, soils were collected from three sites. The contamination analysis was done on these samples through a series of laboratory tests. Furthermore, laboratory tests simulating field conditions were performed in a soil chamber. In the lab test, three leaching agents, namely fresh water, acidic water and seawater, were used. The soil samples used were sand and silt with a relative density of 40% and a compaction ratio of 90%, respectively. The pH of the effluent discharged from the experimental soil chamber was also analyzed. After leaching, soil samples were subjected to analysis. The results showed that pH was higher in the silt than in the sand. The results of the laboratory tests exhibited that leaching of hazardous elements from FNS is limited, so that it can be used as a substitute for natural aggregate in the cement industry or construction applications.

## 1. Introduction

### 1.1. Background and Objective

The iron and steel industry consumes a large volume of raw material and energy for producing steel and discharges various types of byproducts. The volume of byproducts and waste is as much as half the amount of steel produced. Therefore, it is necessary to reduce discharged waste from iron and steel and to reduce the costs for disposing waste by recycling it while avoiding environmental pollution.

In particular, the volume of natural aggregate required for large-scale reclamation (*i.e*., filling the river or the sea with soils and rocks for constructing housing or industrial complexes) is enormous. Therefore, to preserve natural resources, which continue to be depleted, it is essential to maximize recycling byproducts and slag from steel manufacturing as a substitute for natural resources. Recently, some researchers (Komnitsas and Zaharaki [1], Komnitsas [2]) have been trying to improve the technology by which steel wastes and byproducts are recycled.

The total volume of Korea’s waste sharply jumped from 10 million tons in 1996 to 40 million tons in 2001 and is predicted to be more than 0.1 billion tons in 2013. It is known that one million tons of FNS (ferronickel slag) are discharged every year, and FNS can be used for concrete aggregates and fill materials. FNS has high potential as a filling material and concrete aggregate, because it is chemically stable (Wang and Thompson [3], Wang [4], Colangelo and Cioffi [5], Ali *et al*. [6]). Accordingly, other countries, for example, New Caledonia and Japan, started to study FNS 120 years ago and have constructed practical systems through research. Japan established a national standard (Japan Industrial Standard (JIS)) for FNS aggregate to maximize industrial use and recycles almost 100% of the FNS currently discharged (Lee [7]). Because Korea’s recent study of FNS is still at the beginning stages, there is an urgent need to study the basic physical properties of FNS, to identify the chemical mechanism and to develop its use as an aggregate and reclamation material. In spite of the depletion of natural aggregate in Korea, large-scale construction is planned in the Yeosu Industrial Complex near Gwangyang Port and Hadong District, which need a large volume of inert material. Therefore, if FNS is used at these sites, it is necessary to evaluate the effect of FNS for reclamation on the environment, because a lot of variables should be considered, including the evaluation of environmental pollution of underground water depending on precipitation, the effect of FNS on the surrounding ground by soil contamination and environmental contamination due to sea water and fresh water.

Therefore, the objective of the study is to evaluate the engineering properties of FNS and to evaluate the environmental effects of FNS use for reclamation works on surrounding soils. In order to investigate if there is any adverse effect to surrounding soils due to FNS use, soil samples were collected from three sites, and laboratory tests in a soil chamber considering field conditions were performed. Based on the test results, the applicability of FNS as a reclamation material was evaluated.

### 1.2. Previous Studies in Korea and Other Countries

Most studies in Korea and other countries on FNS have focused on the evaluation of the basic performance of concrete. In Japan, a factory for producing ferronickel started to operate in the 1950s and three companies are currently in operation to enable ferronickel slag to be used in various fields. Studies of using ferronickel slag as a high value-added material are under way. In particular, current popular studies include dissolving byproducts and sediment, of which the main components are MgO–SiO_2_ and 2MgO·SiO_2_, to obtain Mg ions.

#### 1.2.1. Previous Studies in Other Countries

In Japan, the volume of FNS was 3.00 million tons in 2013 (Yuki *et al*. [8]). Most FNS has been used as a material for reclamation and road beds. While a fine FNS aggregate was added to the standard JIS A 5011 for slag aggregate for concrete in 1992 to develop guidelines for concrete construction with fine FNS aggregate in 1994, FNS has been used as a fine aggregate for concrete (Kim [9]). In particular, the guideline for recycling technology in port works includes FNS as a recycled material to use it as concrete, filling material and aggregate for road beds and to seek a method of using slag as a resource on a national basis (Masayasu [10]).

According to the Mining Association of Japan, the average specific gravity of fine FNS aggregates used for concrete ranges between 2.78 and 3. The specific gravity of entire FNS samples ranges between 2.73 and 3.13. This is greater than the specific gravity of natural fine aggregate, and its absorption rate is relatively low. It is shown that FNS aggregate is a feasible material for concrete other than freeze-thaw resistance and alkali silica reaction, which is shown in some fine aggregate.

Oelkers [11] pointed out that the forsterite structure is bonded to the silica tetrahedrons by means of magnesium octahedrons, and the bond Mg–O in this structure is more easily broken than the bond Si–O in acid solutions. He also insisted that decomposition of the octahedron bond of Mg can be facilitated by hydrogen ions, and this decomposition contributes to the final decomposition of crystals. That is, Mg ions and Si and SiO_2_ are extracted on the basis of the decomposition of Mg.

Pokrovsky and Schott [12] proved that various ions, including Mg and Si ions, are concurrently extracted if forsterite is dissolved in acid. Therefore, it is necessary to develop various methods of treatment after dissolution in order to obtain Mg ion or Si ion, respectively. However, there is no method of using FNS to produce only Mg ion or Si ion independently.

#### 1.2.2. Previous Studies in Korea

Park *et al*. [13] evaluated the characteristics of concrete mixed with air-cooled slag in order to develop a guideline for concrete by using air-cooled slag in relation to FNS. The evaluation revealed that the amount of added admixture to obtain a required slump increased as the air-cooled slag mix rate increased, resulting in the slightly lower performance of concrete mixed with air-cooled slag. However, they say that the coefficient of elasticity is higher than 90% of typical concrete, suggesting the potential of FNS air-cooled slag as a substitute for fine aggregate.

Kim *et al*. [14] evaluated the performance of fine FNS aggregate and the basic performance of concrete to which FNS was applied for each strength. The evaluation of concrete performance revealed ideal fluidity and compressive strength in comparison with the target quality. They suggested that future application of fine FNS aggregate concrete can be implemented through a performance review for each strength of concrete to which the fine FNS aggregate is applied, as well as field application.

Kim *et al*. [15] evaluated the performance of fine FNS aggregate and the basic performance of concrete to which FNS was applied for each strength in order to develop concrete to which fine FNS aggregate is applied. The evaluation revealed increased viscosity and poor performance when the mix rate was more than 40% in all mixtures of general strength, high strength and super-high strength. Therefore, they concluded that the correction of the granularity and grain shape is needed, and the required unit quantity is reduced as the mix rate is higher, because the absorption rate of FNS is low.

Chu *et al*. [16] extracted Mg and other ions, which are some of the main components of FNS in acid solution, and separated Mg ion from extracted ions to produce a compound thereof. They identified that Mg ions were not the only ions extracted from dissolved slag, but other components were also extracted. Therefore, selective extraction of Mg ion was needed, and they identified that the best extraction condition of Mg ion was at 30 °C for 30 min. Most Si and Fe ions, which exist together with Mg ions, were removed through the filtering process and by adding ammonia water. The compounds, MgCl_2_, NH_4_Cl·6H_2_O, were obtained by drying the final filtered solution, and the crystal MgO was obtained by calcinations of dried material at 600 °C for 30 min.

Most previous studies in Korea and other countries were related to using the engineering characteristics of FNS to use it as a substitute for fine aggregate and fine aggregate used together with concrete. The effect of FNS on the environment considering field conditions has not been studied extensively. Therefore, soil samples at the three sites that were reclaimed near FNS (1 m apart from the locations reclaimed with FNS) were collected for analysis.

## 2. Evaluation of Engineering Properties of FNS

### Engineering Properties of Soils

#### Physical and Mechanical Properties of Soils

The sand sample used in this study was taken from the Seomjingang basin, and the silt sample was taken from the road work site in Damyang. Table 1 illustrates the physical and mechanical properties of the samples, including the specific gravity, permeability of sieve #200 and the USCS (Unified Soil Classification System) classification for soil. Table 2 presents the chemical composition of FNS. Nickel ore in an electric arc furnace was treated to produce FNS.

**Table 1:** Comparison of engineering characteristics of FNS (ferronickel slag), sand and silt. Dr: relative density.

Classification	Unit	Result from Test			KS F
		FNS	Sand	Silt	
Specific gravity	-	2.96	2.66	2.44	KS F 2309
#4	%	69.59	99.58	99.57	KS F 2302
#200	%	0.02	1.24	50.70	KS F 2302
USCS classification	-	SP	SW	ML	KS F 2302
Atterberg limit	-	N.P.	N.P.	LL (%):40.2PL (%):N.P.	KS F 2303, 2304
γ_dmax_	g/cm^3^	1.852	1.710	-	-
γ_dmin_	g/cm^3^	1.547	1.307	-	-
Compaction test γ_dmax_	g/cm^3^	1.893	1.782	1.633	KS F 2312
OMC	%	5.52	11.50	19.10	KS F 2312
Coefficient of permeability (Dr 60%)	cm/s	8.72 × 10^−2^	2.72 × 10^−2^	-	KS F 2322
Ø (Dr 60%)	°	38.66	35.94	28.81	KS F 2343
c	kPa	40.0	29.7	51.8	KS F 2343

KS F: Korean Industrial Standard; SP: poorly graded sand; SW: well graded sand; LL: liquid limit; PL: plastic limit; N.P.: Non plastic; *r*_dmax_: maximum dry unit weight; *r*_dmin_: minimum dry unit weight; OMC: optimum moisture content; ML: Silt.

**Table 2:** Chemical composition of FNS.

Chemical formula	Content (%)
SiO_2_	55.6
MgO	27.8
FeO	7.57
Fe	0.65
CaO	5.18

**Figure 1:**
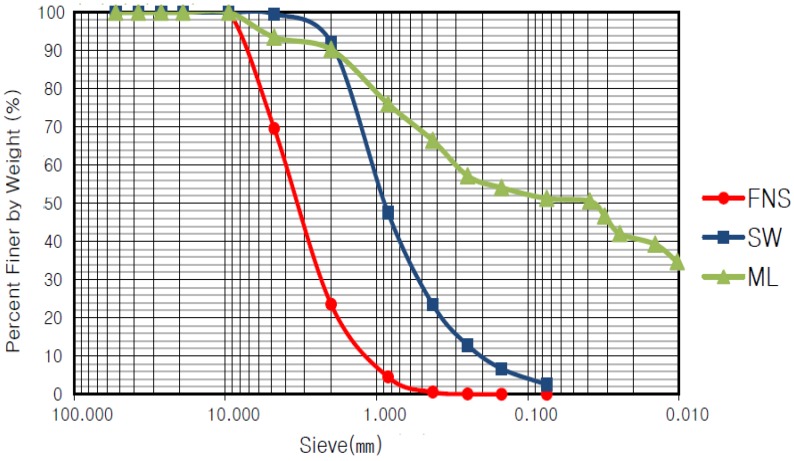
Particle size distribution curve of the samples.

Figure 1 shows the particle size distribution curves of the soils used in this study. The FNS was classified as SP (which means poorly graded sand), as the coefficient of uniformity, Cu, is 3.04 and the coefficient of curvature, *C*_g_ is 1.21. The soil taken from the Seomjingang basin was classified as SW (which means well-graded sand), as its Cu is 6.05 and *C*_g_ is 1.23. The soil taken from the road work site in Damyang was classified as silt and had a liquid limit (LL) of 40.2% and a plastic limit (PL) of N.P. to be classified as an ML sample.

## 3. Evaluation of the Effect of FNS on the Environment at Three Locations

The analysis of original soil contamination aims to identify the effect of FNS on the surrounding soil environment after using FNS for the horizontal drains in the completed Gwangyang Industrial Complex, the construction of the sidewalk of the Yeosu Oil and Chemical Pier and the Suncheon Lakehills Front Plaza. The three sites are near each other (about 50 kilometers away) and located in the southern part of Korea.

### 3.1. Rainfall Condition in Three Locations

According to the Korea Meteorological Administration [17], it is reported that the average monthly rainfall is around 300 mm and the maximum rainfall per hour is, on average, 93 mm in the Chonnam region. Table 3 illustrates the period from the date of completed construction to February 2013, which is the time of sampling for the soil contamination analysis of the original ground (Industrial Complex, Oil and Chemical Pier, Lakehills, TX, USA). Table 4 also illustrates monthly average rainfall in 2012 in Suncheon.

**Table 3:** Construction period, days elapsed and total rainfall in the area for the original ground sampling.

Original Ground	Date of Construction	Days Elapsed (month)	Monthly Average Rainfall (mm)	Total Rainfall (mm)		
				month	day	h
Construction of horizontal drain in Gwangyang Industrial Complex	Mar. 2011–Sep. 2011	17	158	2686	90	4
Current sidewalk in Yeosu Oil and Chemical Pier	Nov. 2011–Sep. 2012	5	152	760	25	1
Suncheon Lakehills Front Plaza	Oct. 2011–Nov. 2011	15	164	2460	82	3

### 3.2. FNS Construction Cases

Table 4 presents the detailed applications of FNS for three sites. FNS was applied to the industrial complex to identify and evaluate a field application as a horizontal drain mat used in vertical drains for reinforcing soft ground. FNS was applied to the sidewalk in the Oil and Chemical Pier to identify the usability of FNS as a road bed material instead of sand. In the Lakehills Front Plaza, FNS was used as a horizontal drain material and fertilizer, because a sub-base and lawn were constructed after laying FNS.

**Table 4:** Detailed use of FNS.

Classification	Horizontal Drain in Gwangyang Industrial Complex	Sidewalk in the Yeosu Oil and Chemical Pier	Suncheon Lakehills Front Plaza
Date of construction	Mar. 2011–Sep. 2011	Nov. 2011–Sep. 2012	Oct. 2011–Nov. 2011
Field sampling	16 Feb. 2013	18 Feb. 2013–22 Feb. 2013	18 Feb. 2013–22 Feb. 2013
Volume of laid FNS	Approximately 26,604 t	Approximately 2,570 t	Approximately 1,525 t
Area for laying FNS	Approximately 2,500 m^2^ (height: 0.3 m)	Approximately 4,630 m^2^ (height: 0.3 m)	Approximately 1,650 m^2^
Rainfall	Approximately 4 mm/h	Approximately 1 mm/h	Approximately 1 mm/h

**Figure 2:**
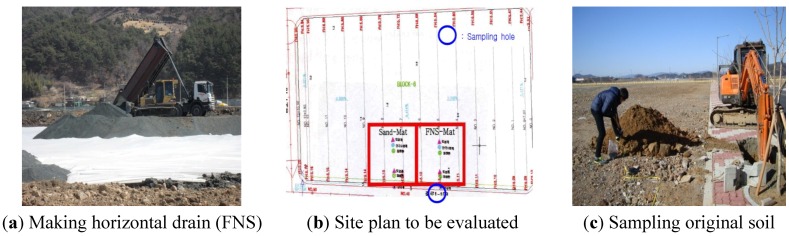
Making the horizontal drain at the Gwangyang industrial Complex and sampling.

In the Gwangyang Industrial Complex site, the FNS was used for constructing the horizontal drains. Figure 2a shows the application of FNS on top of the geotextile. Figure 2b presents a schematic of the two locations where the sand mat and the FNS mat were used. Figure 2c exhibits the sampling of the original soil. The original soil is the soil that has been affected by the FNS due to rainfall.

**Figure 3:**
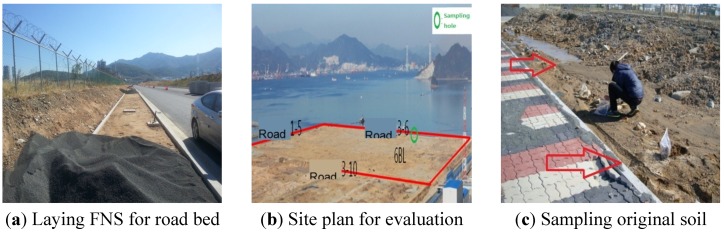
Construction of FNS and sampling of the original soil at the sidewalk at the Yeosu Oil and Chemical Pier.

For constructing a sidewalk in the Yeosu Oil and Chemical Pier for field testing, shown in Figure 3a–c, FNS for road beds was laid on top of the sub-base layer.

**Figure 4:**
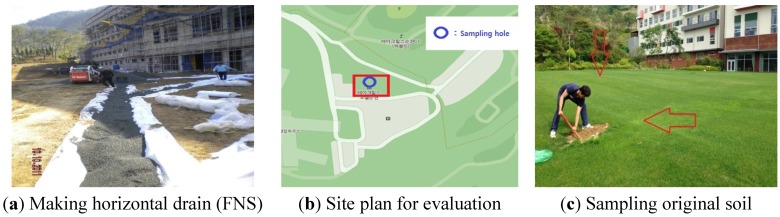
Construction of the FNS and sampling of the original soil at the front plaza of the Suncheon Lakehills Golf Club.

For constructing the Suncheon Lakehills Front Plaza for the field test, Figure 4a shows the application of FNS for the horizontal drain. Figure 4b presents two locations where soil sampling and FNS sampling were done. Figure 4c shows the sampling of the original soil (Choi *et al*. [18], Choi *et al*. [19]).

### 3.3. Results of Analysis of Original Soil

The original soil sample from each area was taken to do soil analysis. Soil analysis was done for seven elements, including As, Cd, Ni, Cu, Zn, Pb and Hg.

Table 5 illustrates the results of the original soil analysis. The results of soil analysis exhibit a high level of Zn in the Industrial Complex, which results from more clay components in the area contributing to the high Zn level. Referring to the study done by Komitsas and Modis [20], the amounts of As and Zn in the soil are similar to the mean values obtained for Russian, Canadian and Netherland soils. The amount of Ni in the soil is much lower than that of Ni in the soils from those countries.

The Oil and Chemical Pier was shown to be relatively stable in comparison with other areas. Case 3 for Suncheon Lakehills is for the original sample, where FNS was laid. This area showed higher levels of As and Ni in comparison with other areas. It is thought that other chemical fertilizers are applied to grow grass, resulting in higher levels than other areas. However, heavy metals detected from the original soil at three locations lie within the allowable range of criteria for the concerned Area 1 for action, and this implies that there is almost no heavy metal contamination in the surrounding soil due to FNS application.

**Table 5:** Results of original soil analysis.

Classification	Original ground	As (mg/kg)	Cd (mg/kg)	Ni (mg/kg)	Cu (mg/kg)	Zn (mg/kg)	Pb (mg/kg)	Hg (mg/kg)
1	Gwangyang Industrial Complex	4.903	0.000	0.000	13.807	138.548	37.874	-
2	Yeosu Oil and Chemical Pier	0.220	0.000	16.202	28.871	100.450	10.693	-
3	Suncheon Lakehills	47.215	0.000	34.279	47.00	134.372	36.716	-
Criteria	Criteria of concern/action for Area 1 (mg/kg)	(25/75)	(4/12)	(100/300)	(150/450)	(300/900)	(200/600)	(4/12)
	Criteria of concern/action for Area 2 (mg/kg)	(50/150)	(10/30)	(200/600)	(500/1500)	(600/1800)	(400/600)	(10/30)
	Criteria of concern/action for Area 3 (mg/kg)	(200/600)	(60/180)	(500/1500)	(2000/6000)	(2000/5000)	(700/2100)	(20/60)

Area 1: dry fields and paddy fields, orchards, dairy farmland, mineral spring sites, building lots (residential), school sites, ditches, fish farms, parks, historical sites and sites for a children’s playground; Area 2: forests and fields, salt farms (other than Area 1), warehouse sites, rivers, river flow management sites, water supply sites, sites for physical exercise, amusement parks, sites for religious purpose, miscellaneous land; Area 3: sites for factories, parking lots, sites for service stations, roads, sites for railroads, banks, miscellaneous land (other than Area 2) and sites for military and national defense facilities.

## 4. Evaluation of Effect of FNS on Neighboring Soils through Soil Chamber Testing

Although the field tests at three sites showed that the effect of FNS on the adjacent soil is minimal, there are many different cases other than the three cases. In order to account for many factors regarding FNS in a more controlled environment, a soil chamber was manufactured. The test in the soil chamber aimed at evaluating if FNS induces any contamination around the reclamation ground in order to use FNS as a reclamation material at a large scale. This test also aimed to identify influential factors in various ways in order to determine the applicability as a reclamation material at a large scale, although it has been used locally to improve soft ground. This test further aimed to conduct component analysis for soil contamination. In this study, FNS was used instead of natural aggregate used as a reclamation material, for example, sand or silt, and rainwater was then made to flow through it. Soil contamination was then analyzed in order to evaluate the effect of FNS on surrounding soil due to rainwater in terms of the environment.

### 4.1. Rainfall Conditions of Soil Chamber Testing

The soil layers in the soil chamber were made of 40 cm-thick original ground, 20 cm-thick FNS and 20 cm-thick soil cover from bottom to top. As it was hard to keep a constant water level, the test was conducted by filling the experimental soil chamber with water to its top and then refilling it with water to its top after a given period of time. Approximately 50 L of discharged effluent were measured in this case, and inverse calculation contributed to selecting the rainfall condition of 2.05 mm/h. Table 6 shows the rainfall condition used in the model test. The rainfall intensity was 4 mm/h, 1 mm/h and 3 mm/h for Gwanyang, Yeosu and Suncheon, respectively. The rainfall intensity is similar to that observed at the three sites.

**Table 6:** Rainfall conditions of soil chamber testing.

Classification	Large chamber (for 5 days)
*Q* (L/day)	50
*A* (cm^2^)	2025
Equation	*Q*/(5 day × 24 h × *A*)
Rainfall intensity (mm/h)	2.05

### 4.2. Mix Ratio of Test Sample

The mix ratio of the experimental soil chamber used in this test was obtained as *e*_max_ = 1.035, *e*_min_ = 0.556 by referring to the relative density test method for cohesionless soil KS F (Korean Industrial Standard) 2345 for sand, to select the amount of dried sample used in the test. Three-layer compaction was conducted to match the sample height to reproduce the ground condition of the target relative density (Dr) of 40%. For silt, *r*_dmax_ = 1.633 was obtained through the compaction test of soil KS F 2312. The amount of sample with a compaction ratio (R) of 90% was selected to match the sample height and then to conduct three-layer compaction in order to reproduce the ground condition of a target compaction ratio (*R*) of 90%.

For FNS, *e*_max_ = 0.913 and *e*_min_ = 0.598 were obtained according to KS F 2345, as for the sand sample, to select the amount of sample used in this test illustrated in Table 7. Fresh water as the leaching agent used in the test was underground water, and HCl solution was used as an acid solution to establish the initial pH as 5.5. For seawater, a natural seawater salt mix was used to produce a leaching agent with conditions similar to seawater.

**Table 7:** Combination ratio of the small and large chamber tests.

Chamber test	Condition		Soil (kg)	Water (L)	Remarks
W 450 × B 450 × H 1000 (mm)	Fresh water	S-2-Dr40	84.23	50	-
		M-2-R90	163.3	50	
	Acidic water	S-2-Dr40	84.23	50	HCl 25 mL
		M-2-R90	163.3	50	
	Seawater	S-2-Dr40	84.23	50	Seawater salt 1800 g
		M-2-R90	163.3	50	

### 4.3. Lab Testing for Soil Contamination Analysis

**Figure 5:**
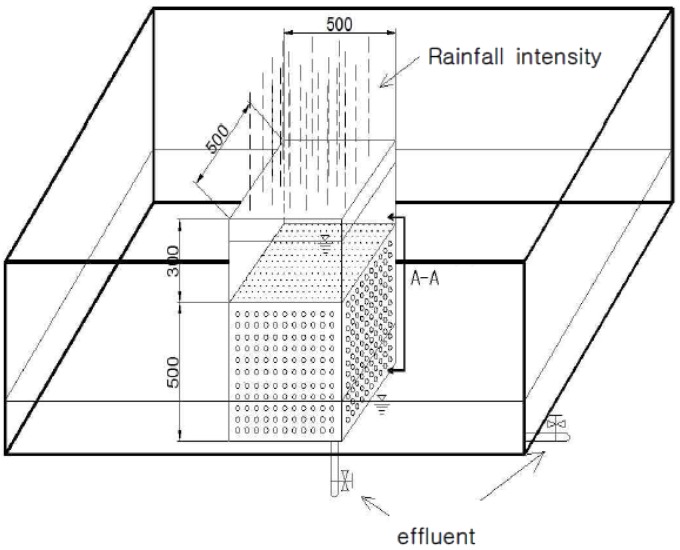
Manufacturing a chamber for testing.

Figure 5 shows the experimental soil chamber, which is divided into an internal soil chamber and an external soil chamber. The internal soil chamber is made of acrylic with dimensions of W 450 mm × B 450 mm × H 1000 mm, and the external soil chamber is made of rubber with dimensions of W 1300 mm × B 700 mm × H 600 mm. The internal soil chamber used free drainage. The rubber soil chamber, which is an external experimental soil chamber, did not have an outlet, and a hole was artificially made to drain flowing effluent. Furthermore, wetting was homogeneous, and no preferential flows were created within the soil mass.

#### Testing with the Experimental Soil Chamber

**Figure 6:**
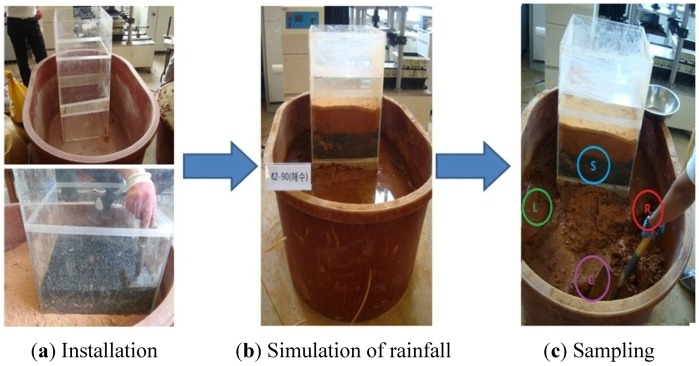
Method for the large chamber test.

The sample selected in Table 7 was made to be in free fall in the experimental soil chamber shown in Figure 6a. Three-layer compaction for sand was then conducted to establish a relative density (Dr) of 40%. For silt, the ground condition was reproduced to be a compaction ratio (*R*) of 90%.

Because the experimental soil chamber did not experience initial pH condition change in fresh water, a constant water level was kept for the test.

If the leaching agent was acid, it was necessary to establish the initial pH as 5.5. Because seawater salt should be mixed to produce seawater, testing could not be carried out in a constant water level.

Therefore, for the rainfall condition, the chamber was fully filled, as shown in Figure 6b, waiting until the water level fell to specify the initial acid and seawater condition. The chamber was filled to the specified level.

Liquid extracted per day was measured with a pH gauge to analyze the effluent from the large experimental soil chamber.

For soil contamination, S in the samples taken from four locations was placed in the lower part of FNS in the internal acrylic chamber shown in Figure 6c. L is the sample placed on the left of the external chamber, R on the right thereof and C in the center. The samples were extracted to dry them at 121 °C with a dryer for soil contamination analysis.

### 4.4. Results of Soil Contamination Analysis through the Experimental Soil Chamber Test

#### 4.4.1. Results of Soil Contamination Analysis for the Original Ground Sample Used in the Experimental Soil Chamber

The soil contamination analysis is based on the criteria of the concerned Areas 1, 2 and 3 specified in the legislation for survey, waterway investigation and land registration. Table 8 lists the results of soil contamination analysis. The original ground sample means a sample in its original state not used in any experimental soil chamber test before this test, and the soil contamination analysis of FNS, sand and silt is described below.

FNS was obtained from Hyoseok Co. in Gwangyang, Korea and showed a bit higher level of As in the soil contamination analysis, which lies within the allowable range of 25 mg/kg for the concerned Area 1. This FNS was thus analyzed without concern regarding contamination. The sand sample for the original ground sample was taken from Okgwa, the Seomjingang basin and an SW (Well graded sand) sample. The soil contamination analysis revealed all compatible levels, and it is thus a sample without concern with regard to contamination.

The silt sample for the original ground sample was taken from the road work site in Damyang, and the soil contamination analysis revealed a very high level of Zn and a great amount of Pb. However, the levels were within the allowable range of 300 mg/kg and 200 mg/kg, respectively, for the concerned Area 1, and thus, implied a sample without concern with regard to contamination.

**Table 8:** Results of soil pollution analysis.

Classification		Sample	As	Cd	Ni	Cu	Zn	Pb	Hg
Sample	1	FNS	21.08	0.00	1.97	1.72	27.04	3.01	0.0051
	2	Sand	8.53	0.00	1.53	1.23	19.13	15.30	0.0231
	3	Silt	14.80	0.41	2.97	3.67	135.87	40.93	0.0180
Sand fresh water	6	S2-40-Fresh (S)	6.68	0.02	1.48	1.75	24.32	12.30	0.0058
	7	S2-40-Fresh (L)	12.16	0.01	5.45	4.60	22.45	15.16	0.0051
	8	S2-40-Fresh (R)	5.04	0.00	1.67	1.81	12.67	10.77	0.0223
	9	S2-40-Fresh (C)	8.49	0.01	2.02	2.25	23.99	15.62	0.0033
Sand acidic water	12	S2-40-acidic (S)	12.48	0.00	1.20	2.66	33.69	24.74	0.0043
	13	S2-40-acidic (L)	14.09	0.00	2.43	1.91	33.68	22.03	0.0040
	11	S2-40-acidic (R)	13.84	0.01	2.45	1.88	31.59	32.85	0.0158
	12	S2-40-acidic (C)	12.17	0.00	1.52	1.89	31.44	17.51	0.0072
Sand seawater	15	S2-40-Sea (S)	9.27	0.12	1.38	1.64	20.34	46.78	0.0045
	16	S2-40-Sea (L)	8.18	0.04	1.44	1.58	24.40	16.65	0.0058
	17	S2-40-Sea (R)	11.85	0.00	2.35	1.52	25.86	18.70	0.0188
	18	S2-40-Sea (C)	6.53	0.01	1.45	1.00	14.14	13.60	0.0223
Silt fresh water	20	M2-90-Fresh (S)	11.76	0.14	0.89	1.57	133.32	12.82	0.0241
	21	M2-90-Fresh (L)	13.32	0.16	1.22	2.11	173.43	13.94	0.0184
	22	M2-90-Fresh (R)	11.80	0.15	1.23	1.98	137.59	14.85	0.0231
	23	M2-90-Fresh (C)	11.91	0.13	1.09	1.86	136.75	13.98	0.0159
Silt acidic water	25	M2-90-acidic (S)	14.88	0.43	3.79	4.29	145.17	47.63	0.0159
	26	M2-90-acidic (L)	15.65	0.45	2.59	4.04	116.59	44.00	0.0072
	27	M2-90-acidic (R)	14.89	0.46	3.21	6.38	125.51	45.21	0.0058
	28	M2-90-acidic (C)	16.96	0.46	5.01	4.48	170.76	44.79	0.0043
Silt seawater	30	M2-90-Sea (S)	15.44	0.41	3.42	4.39	143.26	43.26	0.0051
	31	M2-90-Sea (L)	15.48	0.42	3.21	4.51	131.04	41.61	0.0223
	32	M2-90-Sea (R)	16.13	0.42	3.62	4.16	138.58	42.99	0.0045
	33	M2-90-Sea (C)	16.38	0.41	3.21	4.01	141.13	41.96	0.0033
Standard		Concern/action 1st location (mg/kg)	(25/75)	(4/12)	(100/300)	(150/450)	(300/900)	(200/600)	(4/12)
		Concern/action 2nd location (mg/kg)	(50/150)	(10/30)	(200/600)	(500/1500)	(600/1,800)	(400/600)	(10/30)
		Concern/action 3rd location (mg/kg)	(200/600)	(60/180)	(500/1500)	(2000/6000)	(2000/5000)	(700/2100)	(20/60)

#### 4.4.2. Result of Soil Contamination Analysis of the Experimental Soil Chamber for Sand in Fresh Water, Acidic Water and Seawater Conditions

**Figure 7:**
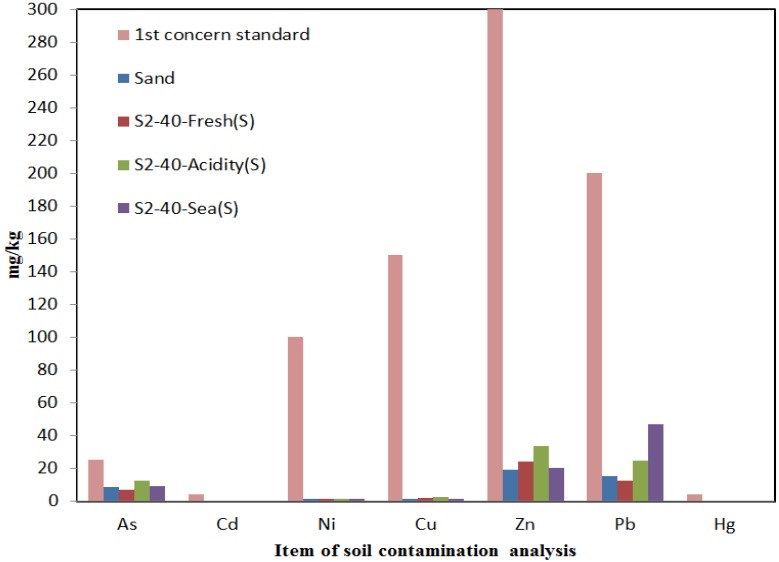
Comparison of the original ground with the standard concerned Area 1 by the leaching agents.

Figure 7 shows the results of the soil contamination analysis of the original ground sample of sand and the sample of Dr 40% of sand used in the experimental soil chamber for the leaching agents of fresh water, acidic water and seawater.

Generally, in the soil contamination analysis for the sandy ground, depending on the leaching agent, a lot of soil contamination occurred for the sand by the leaching of acidic water. This implies acidic rainwater contributes to a high level of contamination. With the fresh water, the level of contamination was the lowest. Therefore, in the sand, the highest contamination level was with the acidic water followed by seawater and then freshwater.

#### 4.4.3. Results of Soil Contamination Analysis of Experimental Soil Chamber for Silt in Fresh Water, Acidic Water and Seawater Conditions

**Figure 8:**
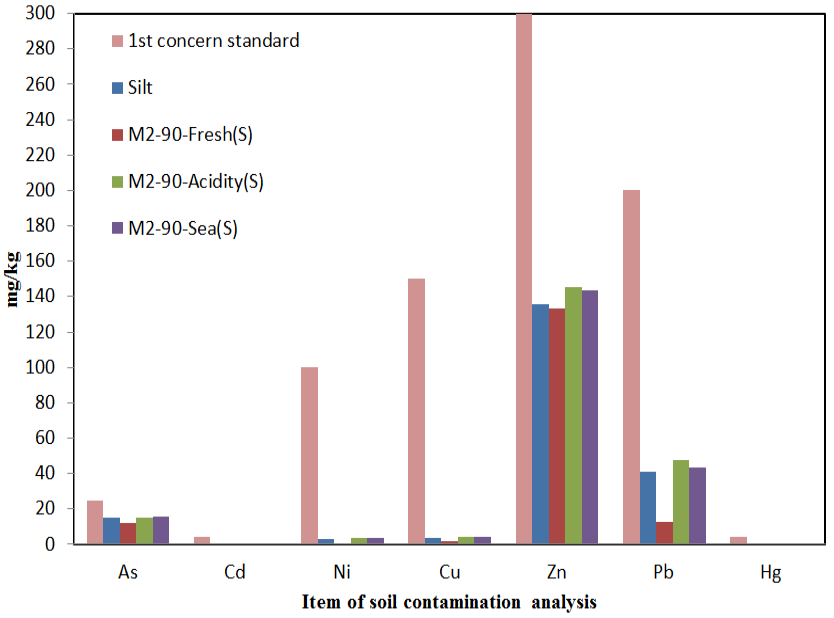
Comparison of the original ground with the standard concerned Area 1 by the leaching agents.

Figure 8 shows the result of soil contamination analysis of the original ground sample for silt and the silt R 90% sample used in the experimental soil chamber test about the effect of the injected liquid of fresh water, acidic water and seawater.

The results of soil contamination analysis of the silt ground, depending on the injected liquid, revealed the lowest level in the fresh water for the silt and a relatively high level in the acidic water as for the sand. Therefore, the highest level of contamination in silt was by acidic water followed by seawater and fresh water.

## 5. Conclusions

The major objective of the study was to analyze the effect of FNS on soil quality and water contamination in the ground where FNS is used. This analysis was done through soil analysis on the soils collected from three sites and from the soil chamber FNS used. An experimental soil chamber was produced in conditions similar to the locations to evaluate the hazardous effects on soil contamination to analyze the effect of FNS on the surrounding soil in terms of the environment. The following conclusions were obtained from this study.

1 The internal friction angle of FNS was measured to be 38.66° for a relative density of 60%, which is approximately 3° greater than that of the sand. This implies that FNS can be used for engineering material instead of sand.
2 The hydraulic characteristics analysis revealed that the coefficient of permeability of FNS was 8.72 × 10^−2^ cm/s for a relative density of 60% in the constant water level permeability test. This is approximately three-times that of typical sand.
3 The analysis of the original ground soil contamination for the sample taken from the Gwangyang Industrial Complex, the Yeosu Oil and Chemical Pier and the Suncheon Lakehills revealed that the detected heavy metals were within the allowable ranges for the concerned Area 1 for action. Therefore, there is almost no concern regarding heavy metal contamination in the surrounding soil by the effluent of FNS one or two years after reclamation with FNS.
4 The analysis of soil with the experimental soil chamber revealed high levels of contamination for both sand and silt with acidic water followed by seawater and fresh water. It was discovered that acid also contributed to contamination. However, there is almost no concern regarding heavy metal contamination in the surrounding soil by the effluent of FNS in reclamation with FNS, because the detected heavy metals were within the allowable ranges for the concerned Area 1 for action.
